# New Inventions

**Published:** 1913-03

**Authors:** 


### NEW INVENTIONS

Martha A. Grafflin and Robt. J. Gregg, Los Angeles, Cal., Syringe, No. 1,046,584.

Bert A. Clayton, Davenport, Ia., assignor to B. J. Palmer, Operating Chair or Table, No. 1,046,736.

Wm. T. Carnes, Kansas City, Mo., assignor to Carnes Artificial Limb Co., Kansas City, Mo., Artificial Arm, No. 1,046,966.

Wm. T. Carnes, Kansas City, Mo., assignor to Carnes Artificial Limb Co., Kansas City, Mo., Artificial Arm, No. 1,046,967.

M. E. G. Gottlieb, Heidelberg-Handschuhsheim, Germany, apparatus for enlarging the air passages of the nose, No. 1,-043,924.

J. H. Drager, Lubeck, Germany, Method of Causing Artificial Respiration, No. 1,044,031.

Frank J. Follansbee, Hope, R. I., Self Conforming Anatomical Truss, No. 1,044,159.

L. T. Little, Homestead, Pa., Jaw Brace, No. 1,044,206.

Ferdinand Cerbo, Providence, R. I., Urethroscope, No. 1,044,-348.

Geo. A. Evans, New York, N. Y., Inhaler, No. 1,044,367.

Moriz Rothenberg, Berlin, Germany, apparatus for use in the Treatment of Fractures of the Thigh or Upper Leg, No 1,044,424.

John B. Campbell, New York, N. Y., Artificial Ear Drum, No. 1,045,812.

Welcome F. Sweet, St. Louis, Mo., Bloat Needle, No. 1,045,-906.

L. P. Valiquet, Newark, N. J., Artificial Ear Drum, No. 1,-045,917.

Fred H. Drake, Oakland, Cali., Capsule, No, 1,046,056.

Wm. E. McNordie, Hamilton, Texas, Surgical Instrument, No. 1,046,098.

J. W. Aylsworth, E. Orange, N. J., Plastic Phenolic Condensation Product, No. 1,046,137.

Per Gsota Ekstrom, Harnes, Sweden, assignor to Oktiebo-

Vincenzo Lazzara, New York, N. Y., Artificial Leg, No. 1,-044,542.

Joseph Payne, Belmont, Mass., Syringe, No. 1,044,688.

Andrew Wineman, Detroit, Mich., Dose-Measuring Bottle, No. 1,044,719.

Walter D. Ball, Philadelphia, Pa., Poison-Bottle Stopper, No. 1,044,726.

Franklin H. Bowly, New York, N. Y., assignor to Mary Webb, New York, N. Y., Nipple, No. 1,044,737.

Edmund M. Pond, Rutland, Vt., Surgical-Bandage, No. 1,-044,817.

Jacques Wittlin, Vienna, Austria-Hungry, assignor to Siegfried Schlewinger, New York, N. Y., Antiseptic, No. 1,044,840.

Otto Bolte, Hamburg, Germany, Stethoscope, Hearing-Trumpet, Ear-Trumpet and like Sound-Conducting Instruments, No. 1,044,858.

Jno. W. Reddy, Geneva, N. Y., Nasal Guard, No. 1,044,910.

Eddie A. Bonar, Detroit, Mich., Artificial Leg, No. 1,044,980.

Otto Graul, Ludwigshafen-on-the-Rhine, assignor to Badische Anilin & Soda Fabrik, Ludwigshafen-on-the-Rhine, Germany, Process of Making Chlorinated Hydrocarbons, No. 1,045,139.

A. E. Guedel, Indianapolis, Ind., Obstetrical Anesthetic Machine, No. ,1045,140.

Wm. R. Lee, Rochester, N. Y., Arch Support, No. 1,045,303.

C. A. Ruflin, Louisville, Ohio, Irribating Catheter, No. 1,-045,326.

Vladimer Stanel, Prague, Austria-Hungary, assignor to Chemische Fabric Gedeon Richter, Budapest, Hungary, Process of Making Solid Stable Compounds Containing Hydrogen Peroxid, No. 1,045,451.

A. H. Tatum, New York, N. Y., assignor to Whitall Tatum Co., of New York, N. Y., Nursing Nipple, No. 1,045,456.

A S. Campbell, Medford, Mass., Hot Water Bottle, No. 1,-045,510.

Ceran Gaveau, New York, N. Y., X-Ray Tube Vacuum Controller, No. 1,045, 543.

A. L. Holtzman, East Orange, N. J., Tooth Brush, No. 1,-045,552.

Alfred Loewy, Berlin, Germany, Rupture Truss, No. 1,-045,574.

Joseph Payne, Melrose, Mass., Case for Hypodermic Needles, No. 1,045,607.

Uriah Hoke, Connwall, Pa., Artificial Limb, No. 1,045,707.

Albrecht Thiele, Berlin, Germany, assignor to Chemische Fabric auf Actien (vorm. E. Schering), Berlin, Germany, Ethyl Ester of 6-Methyl-2-Phenyl Quinolin-4-Carbozylic Acid, No. 1,045,759.

Adolphe Houdard, Neufchatel-en-Bray, France, Bed Pan, No. 1,045,793.

W. M. Ingam, Pueblo, Colo., Hospital Appliance, No. 1,-047,231.

Chas. Katzenberger, Hoboken, N. J., Composition of Matter for Making Formaldehyde Candles, No. 1,047,416.

Rufin Steiner, Oberwyl, near Zug, Surtz, apparatus for Fettering Maniacs and Criminals, No. 1,047,457.

J. W. Aylsworth, E. Orange, N. J., Method of Forming Phenolic Condensation Products No· 1,047,484.

Geo. F. Jaubert, Paris, France, Process of Manufacturing Oxygenated Salts, No. 1,047,645.

Chas. H. Tallmadge, Chicago, Ill., assignor to Wm. H. Heath, Buffalo, N. Y., Method of Preforming Division, No. 1,047,854.

